# Sleeping in an Inclined Position to Reduce Snoring and Improve Sleep: In-home Product Intervention Study

**DOI:** 10.2196/30102

**Published:** 2022-04-06

**Authors:** Sharon Danoff-Burg, Holly M Rus, Morgan A Weaver, Roy J E M Raymann

**Affiliations:** 1 SleepScore Labs Carlsbad, CA United States

**Keywords:** snoring, sleep, sleep tracker, snoring tracker, adjustable bed, digital health, health technology, digital tracker, intervention, measurement

## Abstract

**Background:**

Accurately and unobtrusively testing the effects of snoring and sleep interventions at home has become possible with recent advances in digital measurement technologies.

**Objective:**

The aim of this study was to examine the effectiveness of using an adjustable bed base to sleep with the upper body in an inclined position to reduce snoring and improve sleep, measured at home using commercially available trackers.

**Methods:**

Self-reported snorers (N=25) monitored their snoring and sleep nightly and completed questionnaires daily for 8 weeks. They slept flat for the first 4 weeks, then used an adjustable bed base to sleep with the upper body at a 12-degree incline for the next 4 weeks.

**Results:**

Over 1000 nights of data were analyzed. Objective snoring data showed a 7% relative reduction in snoring duration (*P*=.001) in the inclined position. Objective sleep data showed 4% fewer awakenings (*P*=.04) and a 5% increase in the proportion of time spent in deep sleep (*P*=.02) in the inclined position. Consistent with these objective findings, snoring and sleep measured by self-report improved.

**Conclusions:**

New measurement technologies allow intervention studies to be conducted in the comfort of research participants’ own bedrooms. This study showed that sleeping at an incline has potential as a nonobtrusive means of reducing snoring and improving sleep in a nonclinical snoring population.

## Introduction

Snoring is common and has been associated with poor sleep, increased risk of coronary artery disease, depressive disorders, and other health-related problems [1]. Changing one’s sleeping posture has long been known as a way to reduce snoring [2]. This can include repositioning the upper body to an inclined position to open the upper airways, which can be achieved using specialized pillows, wedges, or bed bases.

These approaches have shown some effectiveness in patients with sleep apnea and other disorders [3]. For example, Skinner et al [4] reported mixed results after testing a shoulder-head elevation pillow for the management of obstructive sleep apnea (OSA). More recently, Souza et al [5] showed that head-of-bed elevation using a laboratory bed reduced the severity of OSA without interfering with sleep architecture. Similarly, a study of a bed that automatically lifted the trunk of the user upon detection of snoring found that it was able to reduce episodes of snoring in the laboratory [6].

However, evidence is lacking in nonclinical populations and settings. The accuracy of new unobtrusive sleep and snoring measurement technologies allows intervention studies to be conducted in research participants’ own bedrooms and may contribute new evidence-based knowledge to the field of applied sleep and snoring research. This method of in-home research using innovative digital health tools has an advantage over traditional sleep laboratory studies. It provides insight into the effectiveness of the intervention under real-life conditions, yielding ecologically valid results while still capturing objective data [7].

In this study, using an adjustable bed base to sleep with the upper body at a 12-degree incline was compared to sleeping in a flat position. The 12-degree angle is sufficient to elevate the head while still being comfortable for sleep. A mild degree of head-of-bed elevation, compared to larger angles, is most likely to be well tolerated while still being effective according to laboratory studies [5,6]. The inclined position was hypothesized to reduce snoring and improve sleep. This was measured objectively over 1000 nights of data collected using commercially available trackers as well as by self-report.

## Methods

### Participants

The sample included 25 users of SleepScore Labs technology who self-reported nightly snoring and screened negatively for sleep apnea based on the NoSAS (Neck Circumference, Obesity, Snoring, Age, Sex) screening tool, using a cut-off score of >8 [8], and/or had a BMI <30 kg/m^2^. Users who reported having a partner who snored were excluded. Users with self-reported sleep disorders or other medical conditions (eg, hyperthyroidism) or lifestyle factors (eg, shift work) affecting sleep were excluded. Of the 25 participants, 60% (n=15) were male and the average age was 38 (SD 11.38) years, ranging from 21-62 years.

### Ethics Approval

All participants provided written informed consent, and all procedures were conducted in accordance with the ethical standards of the 1964 Declaration of Helsinki. Western Institutional Review Board-Copernicus Group reviewed the study under the Common Rule and applicable guidance and determined this to be exempt research.

### Study Design and Procedures

A within-subjects pre-post design was used. During the baseline period, participants slept on their own mattress in a flat position for 4 weeks. During the intervention period, their bed base was replaced with the Dr Oz Good Life Adjustable Base Pro (2020 collection, Maven) to allow for sleeping in an inclined position for 4 weeks using their original mattress. Participants used the preprogrammed Anti-Snore position, resulting in an electrical motor elevating the head of the bed to a 12-degree incline prior to sleep. During the entire 8-week study, participants were instructed to record their snoring and sleep nightly and complete questionnaires daily. Data were collected during the same time period across all participants to account for weekday and weekend variation.

### Measurement of Outcomes

Objective snoring was measured using the Do I Snore or Grind app (Version 1.2.4(2); SleepScore Labs) on an Apple iPod touch sixth generation (Model A1574; Apple Inc) with snoring sensitivity set to high and grinding sensitivity set to low. This consumer app captures sounds using the microphone of the mobile device and identifies snoring using artificial intelligence–based algorithms that filter out nonsnoring sounds. The app quantifies snoring duration exceeding the set level of sensitivity and transforms the percentage of the night during which the user snored into a snore score.

Objective sleep was measured with the SleepScore Max (SleepScore Labs), a noncontact monitoring device using respiratory and motion signals to detect sleep. It uses ultralow-power radiofrequency waves to monitor body movement while in bed; this measurement is unaffected by bedding or nightwear. High-resolution magnitude and duration data of gross movements, micromovements, and full breathing cycles are captured and transformed into 30-second epoch sleep stage data (Wake, Light, Deep, rapid eye movement [REM]) using proprietary algorithms. Studies have shown good agreement between this approach and polysomnography [9,10]. Using the 30-second epoch data, standard sleep metrics were calculated. In addition, the following 3 SleepScore Labs proprietary sleep measures reflecting sleep quality were calculated, all ranging from 0-100 and normalized for age and sex using reference values from the meta-analysis of quantitative sleep parameters by Ohayon et al [11]: SleepScore, which is an overall sleep quality metric that includes objectively measured total sleep time, sleep onset latency, and sleep stage durations; BodyScore, which reflects the age- and sex-normalized amount of deep (non–rapid eye movement stage 3 [NREM-3]) sleep; and MindScore, which reflects the age- and sex-normalized amount of REM sleep. The device’s sensor is placed next to the bed and a companion app shows users their sleep data along with insights and advice.

Self-report items were developed for the current study. Perceived snoring (nights per week snored and frequency of waking from snoring) was measured before and after the intervention period and perceived sleep (time to fall asleep, number of awakenings during the night, amount of time spent awake after initially falling asleep, feeling well-rested in the morning, and overall sleep quality) was measured daily. These data were collected on the internet using SurveyMonkey (Momentive Inc).

### Statistical Analyses

Statistical analyses were performed in R (version 3.5.2; R Foundation for Statistical Computing). Nightly objective snoring data, objective sleep data, and self-reported sleep data were analyzed using multilevel regression with a random intercept model, accounting for nights nested within participants and comparing nights during the baseline period to nights during the intervention period for each outcome. The regression model used was the following: SleepMeasure_ij _= Const_0ij _+ β × TestPeriod_ij_; TestPeriod was coded as 0 for observations during the baseline and 1 for nights during the intervention period. 

Self-reported snoring outcomes were analyzed with paired-samples *t* tests.

Discrepancies in sample sizes (N=1181 for snoring, N=991 for sleep, and N=1185 for self-report) occurred as the data sources were incomplete. Participants tracked their snoring and sleep at home and at times were not fully compliant with using these measurement tools or completing daily surveys on the internet. All results reported reflect the largest sample available for each set of analyses.

## Results

### Snoring-Related Outcomes

Night-to-night objective measurement of snoring (1181 nights nested within 25 participants) revealed a 7% relative reduction in snoring duration (*P=*.001) when sleeping in the inclined position compared to the flat position (see Table 1).

Similar to the objective findings, self-report data indicated that participants felt they snored less often (decreasing on average from 6 nights per week to 5; *P=*.01) and were woken up by their snoring less often (decreasing on average from sometimes to rarely; *P<*.001) in the inclined position compared to the flat position. Participants with a bed partner (n=10) reported that their partner woke them less often to stop snoring (decreasing on average from sometimes to rarely*; P<*.01) when sleeping in the inclined position.

**Table 1:** Objective snoring and objective sleep multilevel regression results comparing the baseline to the intervention period.

Outcomes			Observed mean (SD)^a^					Estimated marginal means^b^				
			Baseline period		Intervention period		Intercept (SE)			β^c^		*P* value
**Objective snoring (1181 nights)**												
	Snore score^d^	9.28 (4.29)		8.61 (3.64)		9.31 (0.24)			–0.756		.001	
**Objective sleep (991 nights)**												
	Time in bed in minutes	452.76 (76.35)		445.66(75.57)		450.24 (5.16)			–4.689		.18	
	Total sleep time in minutes	391.27 (69.05)		383.78(68.16)		388.79 (4.61)			–5.534		.12	
	Sleep efficiency^e^	85.43 (5.66)		84.02(5.92)		86.19 (0.41)			0.137		.37	
	Sleep onset latency in minutes	18.65 (14.81)		18.05(13.23)		18.54 (1.01)			0.095		.46	
	Number of awakenings	4.45 (1.99)		4.25(2.07)		4.43 (0.13)			–0.237		.04	
	Percentage of time spent awake after sleep onset	9.10 (4.87)		9.25(5.52)		9.10 (0.37)			0.032		.47	
	Percentage of time in light sleep	54.30 (7.07)		53.55(6.86)		54.40 (0.46)			–0.668		.07	
	Percentage of time in deep sleep	19.30 (6.55)		20.20(7.44)		19.30 (0.45)			0.905		.02	
	Percentage of time in REM^f^ sleep	17.30 (5.37)		17.00(5.66)		17.20 (0.36)			–0.298		.21	
	SleepScore^g^	77.63 (9.73)		78.27(9.98)		77.40 (0.67)			0.633		.17	
	BodyScore^g^	78.93 (9.59)		81.10(10.13)		78.85 (0.63)			2.109		<.001	
	MindScore^g^	76.99 (12.99)		75.62(14.46)		76.61 (0.93)			–1.421		.06	

^a^For the baseline and intervention periods, each mean was calculated by averaging nights across participants, then averaging those participants’ averages to a single simple average.

^b^These are the outcomes of separate multilevel regression analyses. Each row shows results from a different single-predictor, single-outcome model.

^c^The beta values are unstandardized and can therefore be interpreted on the same scale as the original data.

^d^Snore score is reported as a percentage.

^e^Sleep efficiency is calculated as the ratio of time spent asleep to time spent in bed and reported as a percentage.

^f^REM: rapid eye movement.

^g^These scores range from 0 to 100.

### Sleep-Related Outcomes

Night-to-night objective measurement of sleep (991 nights nested within 25 participants) revealed that when sleeping in the inclined position, participants woke up less often (4% decrease in number of awakenings; *P=*.04) and experienced a greater proportion of deep sleep (5% relative increase; *P=*.02), reflected by an improved BodyScore (3% increase; *P<*.001). Detailed sleep metrics are displayed in Table 1.

Multilevel analyses of the self-reported sleep data (1185 nights nested within 25 participants) showed that participants perceived that they fell asleep faster (20% decrease in sleep onset latency; *P<*.001), woke up less often (15% decrease in number of awakenings; *P=*.001), felt more rested in the morning (17% increase in the score on the 0-100 scale; *P<*.001), and experienced better sleep quality (14% increase in the score on the 0-100 scale; *P<*.001) in the inclined position compared to the flat position (see Table 2).

**Table 2:** Multilevel regression results for self-reported sleep (1185 nights), comparing the baseline to the intervention period.

Outcomes	Observed mean (SD)^a^		Estimated marginal means^b^		
	Baseline period	Intervention period	Intercept (SE)	β^c^	*P* value
Perceived sleep onset latency in minutes	16.38 (11.80)	13.13 (9.35)	16.23 (0.79)	–2.922	<.001
Perceived number of awakenings	2.33 (1.44)	1.97 (1.31)	2.31 (0.10)	–0.320	.001
Perceived amount of time spent awake after sleep onset in minutes	14.10 (14.43)	13.00 (13.36)	14.13 (1.38)	–0.898	.26
Feeling well-rested in morning^d^	56.29 (16.38)	66.09 (16.30)	56.60 (1.08)	9.651	<.001
Perceived sleep quality^d^	58.94 (15.52)	67.47 (16.78)	59.47 (1.06)	8.078	<.001

^a^For the baseline and intervention period, each mean was calculated by averaging nights across participants, then averaging those participants’ averages to a single simple average.

^b^These are the outcomes of separate multilevel regression analyses. Each row shows results from a different single-predictor, single-outcome model.

^c^The beta values are unstandardized and can therefore be interpreted on the same scale as the original data.

^d^These outcomes are reported as a score ranging from 0-100.

## Discussion

Recent technological advances enable the accurate digital measurement of sleep and snoring in the comfort and familiarity of one’s habitual bedroom [12,13]. In this study, participants slept on their own mattresses in their homes for the duration of the study, recording their snoring and sleep nightly with commercially available trackers and by self-report. They slept flat for 4 weeks and then used the Anti-Snore setting (12-degree incline) of the Adjustable Base Pro for 4 weeks.

Analyses of over 1000 nights of data showed a significant improvement across all 4 areas measured: objective snoring, perceived snoring, objective sleep, and perceived sleep. When sleeping with the upper body in the inclined position, compared to when sleeping flat, objectively measured snoring duration decreased and self-reported snoring outcomes improved. Objective sleep measurements revealed fewer awakenings and more time in deep sleep when sleeping in the inclined position. Related to the increase in deep sleep, participants’ average BodyScore increased. Self-reported sleep data showed fewer perceived awakenings and better sleep quality; additionally, participants felt more well-rested in the morning. Participants also felt that they fell asleep faster in the inclined position, but this finding was not confirmed by the objective sleep data. Taken together, these findings suggest that future in-home product intervention research using commercially available snoring and sleep trackers is merited.

In conclusion, sleeping with the upper body at an incline has potential as a simple nonobtrusive means of reducing snoring and improving sleep in a nonclinical snoring population. This sleeping position is thought to lead to benefits by decreasing upper airway collapsibility and increasing the upper airway area, compared to the flat position, leading to improved breathing and, in turn, better sleep [5]. Elevating the upper body at night is also frequently recommended to alleviate heartburn symptoms and improve sleep in individuals experiencing nocturnal gastroesophageal reflux [14]. This nonpharmacologic approach is preferred by many patients. Similarly, snorers might prefer this intervention because it is incorporated into their bedroom furniture, does not require extra pillows or devices, and may provide more comfort than nasal or oral appliances that can reduce snoring.

Study limitations include the need for further validation testing of the commercially available snoring app and the absence of a control group. Applied in-home product intervention studies have limitations compared to clinical trials; the key is to assess any change from no product use to product use as it would be experienced outside of a research setting. The design best reflecting this real-life experience is a baseline-controlled intervention. By using a within-subjects design with long-term use of the intervention, we have confidence that the observed changes are due to the intervention itself.

In this study, data were not collected regarding the sleeper’s preferred body position (eg, supine or lateral) and therefore, possible interactions of sleeping position with the incline were not explored. These interactions have been shown to be relevant in determining optimal positional therapy for sleep apnea [15] and would be an interesting addition to future studies of snorers at home. Another useful future research direction could be measuring the sleep of snorers’ bed partners because the elimination of snoring has been shown to benefit partners’ sleep [16]. These types of research questions can now be studied using digital technology that is commercially available to consumers for home use.

## Abbreviations

NoSAS: Neck Circumference, Obesity, Snoring, Age, Sex screening tool
NREM-3: non–rapid eye movement stage 3
OSA: obstructive sleep apnea
REM: rapid eye movement
